# Supplementary material for: Molecular Networking Leveraging the Secondary Metabolomes Space of Halophila stipulaceae (Forsk.) Aschers. and Thalassia hemprichii (Ehrenb. ex Solms) Asch. in Tandem with Their Chemosystematics and Antidiabetic Potentials
Source: Mar Drugs. 2021 May 18;19(5):279. doi: 10.3390/md19050279 (PMC8157295; doi:10.3390/md19050279)
Supplement: Supplementary file 1 [file marinedrugs-19-00279-s001.zip › marinedrugs-1213609-supplementary.pdf]

Supplementary Data

Molecular Networking Leveraging the Secondary Metabolomes Space of *Halophila stipulaceae* (Forsk.) Aschers. and *Thalassia hemprichii* (Ehrenb. ex Solms) Asch. in Tandem with Their Chemosystematics and Antidiabetic Potentials

Nesrine M. Hegazi[1], Hamada H. Saad[1,2*], Mona M. Marzouk[1], Mohamed F. Abdel Rahman[3], Mahitab H. El Bishbishy[4], Ahmed Zayed[5,6], Roland Ulber[5*], Shahira M. Ezzat [4,7**]**

[1] Department of Phytochemistry and Plant Systematics, Division of Pharmaceutical Industries, National Research Centre, Dokki, Cairo, Egypt. nm.hegazi@nrc.sci.eg & monakhalil66@hotmail.com

[2] Department of Pharmaceutical Biology, Pharmaceutical Institute, Eberhard Karls University of Tübingen, PO Box 72074, Tübingen, Germany. hamada.saad@pharm.uni-tuebingen.de

[3] Department of Biology and Biochemistry, School of Life and Medical Sciences, University of Hertfordshire Hosted by Global Academic Foundation, Cairo, Egypt. m.farouk@gaf.edu.eg

[4] Department of Pharmacognosy, Faculty of Pharmacy, October University for Modern Sciences and Arts (MSA), Giza 12451, Egypt. mahelmy@msa.eun.eg

[5] Institute of Bioprocess Engineering, Technical University of Kaiserslautern, Gottlieb-Daimler-Straße 49, 67663 Kaiserslautern, Germany. ulber@mv.uni-kl.de & zayed@mv.uni-kl.de

[6] Department of Pharmacognosy, Tanta University, College of Pharmacy, El-Guish Street, 31527 Tanta, Egypt. ahmed.zayed1@pharm.tanta.edu.eg

[7] Department of Pharmacognosy, Faculty of Pharmacy, Cairo University, Kasr El-Aini Street, Cairo 11562, Egypt. shahira.ezzat@pharma.cu.edu.eg

***Corresponding author**
Institute of Bioprocess Engineering, Technical University of Kaiserslautern, Gottlieb-Daimler-Straße 49, 67663 Kaiserslautern, Germany
Tel.: +49 (0) 631 205 4043; Fax: +49 631 205-4312

****Co-Corresponding author**
Department of Pharmacognosy, Faculty of Pharmacy, Cairo University, Cairo 11562, Egypt. Department of Pharmacognosy, Faculty of Pharmacy, October University for Modern Sciences and Arts (MSA), Giza 12451, Egypt. shahira.ezzat@pharma.cu.edu.eg; Tel.: +20-120-000-4301

*****Co-Corresponding author**
Department of Phytochemistry and Plant Systematics, Division of Pharmaceutical Industries, National Research Centre, Dokki, Cairo, Egypt. Department of Pharmaceutical Biology, Pharmaceutical Institute, Eberhard Karls University of Tübingen, PO Box 72074, Tübingen, Germany. hamada.saad@pharm.uni-tuebingen.de

Table of contents

Supplementary Tables

Table S1: Distribution and ^{1}H-NMR of the isolated aglycones among *Halophila stipulaceae* (*Hs*) and *Thalassia hempiricii* (*Th*) **4**

Table S2: Compounds assignment of *Halophila stipulaceae* (*Hs*) and *Thalassia hempiricii* (*Th*) extracts as revealed by UPLC-HRMS/MS analysis **5**

Table S3: Results of Enzymes inhibition assays of *Hs* and *Th* extracts **21**

Table S4: Summary of the biomarkers in different study groups of diabetic rats **21**

Supplementary Figures

Fig. S1. HPLC profile of the *Halophila stipulaceae* (*Hs*) extract detected at wavelengths 210, 250, 285, 375 nm. **22**

Fig. S2. HPLC profile of *Thalassia hempiricii* (*Th*) extract detected at wavelengths 210, 250, 285, 375 nm. **22**

Fig. S3. The base peak chromatograms (BPC) of *Halophila stipulaceae* (*Hs*) extract (blue) and *Thalassia hempiricii* (*Th*) extract (red) in the negative ionization mode. **23**

Fig. S4. The base peak chromatograms (BPC) of *Halophila stipulaceae* (*Hs*) extract (blue) and *Thalassia hempiricii* (*Th*) extract (red) in the positive ionization mode. **23**

Fig. S5. Enlarged positive molecular network created using MS/MS data (positive mode) from *Halophila stipulaceae* (*Hs*) (purple nodes) and *Thalassia hempiricii* (*Th*) (yellow nodes). **24**

Fig. S6. Proposed fragmentation scheme and MS^{2} spectrum of methoxy benzoic acid -*O*-sulphate, **16** **24**

Fig. S7. Proposed fragmentation scheme and MS2 spectrum of dimethoxy benzoic acid-O-sulphate, **30**. **25**

Fig. S8. Proposed fragmentation scheme and MS^{2} spectrum of *O*-caffeoyl *O*-hydroxyldimethoxybenzoyl tartaric acid, **26.** **25**

Fig. S9. Proposed fragmentation scheme and MS^{2} spectrum of methoxypentahydroxyflavanone-*O*- hexoside**, 28.** **26**

Fig. S10. Proposed fragmentation scheme and MS2 spectra of acetylated rebaudioside, **96** vs. its non-acetylated ascendant, rebaudioside **;72** **26**

Fig. S11. EIC of syphonoside, **53** in both extracts **27**

Fig. S12. Proposed fragmentation scheme and MS^{2} spectrum of syphonoside, **53** in both extracts **27**

Supplementary Results and Discussion

Compounds annotation, lipids **28**

References **30**

Supplementary Tables

Table S1: Distribution and ^{1}H-NMR of the isolated aglycones among *Halophila stipulaceae* (*Hs*) and *Thalassia hempiricii* (*Th*)

Isolated aglycones	*Hs*	*Th*	^{1}H-NMR (500 MHz in DMSO-d_6, δ, ppm, *J*/Hz)	References
Apigenin	+++	++	12.91(1H, s, 5-OH), 7.91 (2H, d, *J*= 8.7 Hz, H-2′,H-6′), 6.92 (2H, d, *J*= 8.7 Hz, H-3′, H-5′), 6.72 (1H, s, H-3), 6.51 (1H, d, *J* = 2.0 Hz, H-8), 6.2 (1H, d, *J* = 2.0 Hz, H-6)	[1]
Genkwanin	+	t	Co-PC	[2]
Scutellarein	-	++	Co-PC	[3]
Isoscutellarein	-	+++	13.01(1H, s, 5-OH), 7.89 (2H, d, *J*= 8.6 Hz, H-2′,H-6′), 6.88 (2H, d, *J*= 8.7 Hz, H-3′, H-5′), 6.76 (1H, s, H-3), 6.18 (1H, s, H-6)	[3]
Hispidulin	-	+	Co-PC	[4]
Cirsimaritin	++	+	13.01(1H, s, 5-OH), 8.01 (2H, d, *J*= 8.6 Hz, H-2′,H-6′), 6.98 (2H, d, *J*= 8.7 Hz, H-3′, H-5′), 6.85 (1H, s, H-8), 6.73 (1H, s, H-3), 3.83 (3H, s, 7-OCH_3), 3.71 (3H, s, 6-OCH_3)	[5]
Luteolin	++	++	12.98 (1H, s, 5-OH), 7.42 (2H, m, H-2′, H6′), 6.87 (1H, d, *J*= 8.5 Hz, H-5′), 6.65 (1H, s, H3), 6.43 (1H, d, *J*=2.0Hz, H-8), 6.18 (1H, d, *J* = 2.0Hz, H-6)	[1]
Chrysoeriol	++	+	12.96 (1H, s, 5-OH), 7.58 (2H, m, H-2′, H6′), 6.93 (1H, d, *J*= 8.4 Hz, H-5′), 6.75 (1H, s, H3), 6.53 (1H, d, *J*=2.1Hz, H-8), 6.18 (1H, d, *J* = 2.1Hz, H-6)	[1]
6-hydroxyl luteolin	-	+++	13.05 (1H, s, 5-OH), 10.33 (1H, s, 6-OH), 7.44 (2H, m, H-2′, H6′), 6.98 (1H, d, *J*= 8.5 Hz, H-5′), 6.58 (1H, s, H3), 6.43 (1H, d, s, H-8)	[4]
Pedalitin	t	+	Co-PC	[5]

+++; major, ++; strong, +, present, t; trace,-; absent, Co-PC; Comparative paper chromatography with authentic samples

Table S2: Compound assignment of *Halophila stipulaceae* (*Hs*) and *Thalassia hempiricii* (*Th*) extracts as revealed by UPLC-HRMS/MS analysis

No.	Rt	Compound class	Compound Assignment	[M-H]$^-$	[M+CH$_2$O$_2$-H]$^-$	[M+H]$^+$	MS^2 -ve	MS^2 +ve	Molecular formula (error in ppm)	*Hs*	*Th*	References
**1**	5.8	Organic acid	Malic acid [e]	133.0143			115		$C_4H_6O_5$ (0.3)	√	√	[6]
**2**	5.91	Organic acid	Citric acid [e]	191.0197			111		$C_6H_8O_7$ (0.3)		√	Marzouk *et al.*, 2018
**3**	7.7	Amino acid	Tyrosine *O*-sulfate [e]	260.0234			180<br>163<br>146<br>119		$C_9H_{11}NO_6S$ (0.0)	√	√	
**4**	7.95	Organic acid	Succinic acid [e]	117.0194					$C_4H_6O_4$ (0.8)	√	√	[7]
**5**	9.1	Aromatic heterocyclic organic compound	Indoline [e]			120.0810		103	C_8H_9N (1.8)		√	GNPS libraries
**6**	9.16	Amino acid	Phenylalanine [e]			166.0866		120<br>103	$C_9H_{11}NO_2$ (2.59)		√	[8]
**7**	9.48	Fatty acid	Aminooctanedioic acid [e]	188.0930			173<br>117		$C_8H_{15}NO_4$ (0.9)		√	[9]
**8**	9.5	Benzoic acid ester	Vanillic acid-*O*-glucoside isomer [e]	329.0874			209<br>167<br>123		$C_{14}H_{18}O_9$ (0.3)		√	[10]
**9**	9.55	Aromatic heterocyclic organic compound	Tetrahydroxyoctahydro-indolizine [e]	188.0932			170<br>144<br>118		$C_8H_{15}NO_4$ (0.9)		√	Rajana *et al.*, 2018

**10**	9.8	Benzaldehyde	Vanillin-*O*-glucoside [e]	313.0927		151<br>135<br>107		$C_{14}H_{18}O_8$ (0.8)	√	√	[11]
**11**	10.07	Benzoic acid	Syringic acid-*O*-glucoside [c]	359.0982		197<br>153<br>138<br>123		$C_{15}H_{20}O_{10}$ (0.4)	√	√	[12]<br>[13]
**12**	10.08	Cinnamic acid	Hydroxyphenyl-sulfooxypropanoic acid [c] (tichocarpol A) [e]	261.0072		181<br>163<br>135		$C_9H_{10}O_7S$ (0.82)	√		[14]
**13**	10.81	Benzoic acid	Vanillic acid-*O*-glucoside isomer [e]	329.0875		167<br>153		$C_{14}H_{18}O_9$ (1.1)		√	[12]
**14**	11.24	Amino acid	Tryptophan [d]		205.1033		146	$C_{11}H_{12}N_2O_2$ (-4.4)		√	GNPS libraries
**15**	11.3	Aromatic heterocyclic organic compound	Indole Acrylic acid [e]		188.0712		146	$C_{11}H_9NO_2$ (-3.3)		√	
**16**	11.4	Benzoic acid	Methoxy benzoic acid -*O*-sulphate [f]	230.9970		151<br>107		$C_8H_8O_6S$ (2.7)	√	√	
**17**	11.6	Cinnamic acid ester	*O*-Caffeoyl tartaric acid (Caftaric acid) [d]	311.0439	-	179<br>149<br>135	-	$C_{13}H_{12}O_9$ (0.1)		√	[15]
**18**	11.9	Benzoic acid	Protocatechuic acid [d]	153.0194		109		$C_7H_6O_4$ (0.6)		√	[16]
**19**	12.33	Cinnamic acid	Sinapic acid-*O*- glucoside [d]	385.1138		223<br>206<br>179<br>165		$C_{17}H_{22}O_{10}$ (0.5)		√	[17]
**20**	12.33	Cinnamic acid	Coumaric acid-*O*- glucoside [d] (Isomer I)	325.0923		163<br>145<br>119		$C_{15}H_{18}O_8$ (1.7)	√	√	[12]

**21**	12.49	Cinnamic acid	Dihydrocoumaroyl-*O*-glucoside [e] (Isomer I)	327.1081	-	165<br>121	-	$C_{15}H_{20}O_8$ (0.6)		√	[18]
**22**	12.51	Aromatic heterocyclic organic compound	Ethenyl indole [e]	-	144.0811	-	117	$C_{10}H_9N$ (-2.7)		√	GNPS libraries
**23**	12.57	Cinnamic acid	Dihydrocoumaroyl-*O*-glucoside [e] (Isomer II)	327.1083		165<br>121		$C_{15}H_{20}O_8$ (0.6)	√		[18]
**24**	12.81	Cinnamic acid	Coumaric acid-*O*- glucoside [d] (Isomer II)	325.0932		163<br>145<br>119		$C_{15}H_{18}O_8$ (0.9)		√	[12]
**25**	12.9	Flavone	Drymariatin A (rhamnosyl)ethenyl-trihydroxyflavone [e]	-	459.1299	-	283	$C_{23}H_{22}O_{10}$ (3.7)	√		[19]
**26**	13.03	Cinnamic acid	*O*-Caffeoyl *O*- hydroxyl dimethoxy benzoyl tartaric acid [f]	491.0829		329<br>311<br>179<br>149<br>135		$C_{22}H_{20}O_{13}$ (0.9)		√	
**27**	13.15	Flavone	Apigenin di-*O*- glucoside [e]	-	595.1672	-	433<br>271	$C_{27}H_{30}O_{15}$ (2.4)	√		[20]
**28**	13.41	Flavanone	Hexahydroxy-mono-methoxyflavanone-*O*-glucoside [f]	-	513.1254	-	351<br>333	$C_{22}H_{24}O_{14}$ (3.04)	√		[21]
**29**	13.48	Flavone	Chrysoeriol -*O*- diglucoside [e]	-	625.1785	-	463<br>301	$C_{28}H_{32}O_{16}$ (3.51)	√		GNPS libraries [22]
**30**	13.25	Benzoic acid	Dimethoxy benzoic acid-*O*-sulphate [f]	261.0075		181<br>137		$C_9H_{10}O_7$ S (0.0)		√	
**31**	13.3	Cinnamic acid	Ferulic acid-*O*- glucoside [e]	355.1035		193<br>175		$C_{16}H_{20}O_9$ (0.2)		√	[23]

						161<br>135					
**32**	13.5	Cinnamic acid	*O*-Coumaroyl tartaric acid (Coutaric acid) [d]	295.0460	-	163<br>119	-	$C_{13}H_{12}O_8$ (0.1)		√	[15]
**33**	13.9	Flavanone	Tetrahydroxy flavanone-*O*-glucoside [e]	449.1090	-	287<br>269<br>259<br>179<br>149	-	$C_{21}H_{22}O_{11}$ (0.4)		√	[24]
**34**	13.93	Cinnamic acid	Caffeic acid-*O*-sulphate [d]	258.9919		179<br>135		$C_9H_8O_7S$ (-0.2)		√	[25]
**35**	14.27	Benzoic acid	*p*-hydroxy benzoic acid [c,d]	137.0244		109		$C_7H_6O_3$(0.6)		√	[13, 26]
**36**	14.57	Flavone	Dihydroxy dimethoxy flavone-*O*- glucoside [d, e]	-	477.1404	-	315	$C_{23}H_{24}O_{11}$ (3.1)	√	√	[27]
**37**	14.59	Flavone	6-Hydroxyl luteolin -*O*-glucoside [d] (Isomer I)	463.0884	-	301	-	$C_{21}H_{20}O_{12}$ (0.6)	√	√	[28]
**38**	14.7	Cinnamic amide	Dicoumaroyl spermidine [e] (Isomer I)	-	438.2405	-	292<br>204<br>147	$C_{25}H_{31}N_3O_4$ (2.5)	√		[29]
**39**	14.9	Cinnamic acid	Caffeic acid [c,d]	179.0351	181.0502	135	163<br>135	$C_8H_8O_4$ (-0.4)		√	[13]
**40**	15.0	Cinnamic acid	Caffeoyl malic acid [e]	295.0461	-	179<br>133	-	$C_{13}H_{12}O_8$ (-1.4)		√	[30]
**41**	15.03	Coumarin	Esculetin (dihydroxycoumarin) [e]	177.0193	-	149<br>133	-	$C_9H_6O_4$ (0.34)		√	GNPS library
**42**	15.2	Flavone	6-Hydroxyl luteolin -*O*-rutinoside [e]	609.1463	611.1616	301	303	$C_{27}H_{30}O_{16}$ (0.5)		√	GNPS library [31]
**43**	15.37	Cinnamic amide	Dicoumaroyl spermidine (Isomer II) [e]	-	438.2401	-	292,<br>2041	$C_{25}H_{31}N_3O_4$ (3.6)	√		[29]

								47				
**44**	15.4	Benzoic acid	Dihydroxybenzoylmethyl ester-(-*O*-vanilloyl)-xylosyl glucoside [e]	611.1619		-	461<br>311<br>167	-	$C_{27}H_{32}O_{16}$ (0.2)		√	[32]
**45**	15.9	Flavone	Luteolin *O*-glucoside sulphate sodium salt [e]	549.0313			285		$C_{21}H_{19}NaO_{14}S$		√	[33]
**46**	16.0	Flavone	Luteolin 7-*O*-glucoside sulphate<br>(Thalassiolin A) [c]	527.0501		529.0662	447<br>285<br>241<br>151		$C_{21}H_{20}O_{14}S$ (0.16)		√	[33]
**47**	16.13	Flavone	Luteolin-*O*-glucoside [d]<br>(Isomer II)	2.0884			285		$C_{21}H_{20}O_{11}$ (1.6)	√	√	[34]
**48**	16.13	Flavone	6-Hydroxyl luteolin -*O*-glucoside [d]	463.0878		465.1036	301<br>271	303	$C_{21}H_{20}H_{12}$ (-0.2)		√	[31]
**49**	16.7	Flavone	Luteolin-*O*-glucoside [d]	447.0938		44089	285	287	$C_{21}H_{20}O_{11}$ (0.7)	√		Enerstvedt *et al.* 2016)
**50**	16.2	Flavone	Luteolin [d]			287.0557		151	$C_{15}H_{10}O_{6}$ (-4.8)	√		[28]
**51**	16.24	Flavone	Pedalitin-*O*- glucoside [e]	477.1038		479.1195	315<br>300	317	$C_{22}H_{22}O_{12}$ (0.08)	√	√	GNPS libraries [35]
**52**	16.3	Flavanone	Pentahydroxy flavanone-*O*-glucoside [e]	465.1036			303		$C_{21}H_{22}O_{12}$ (2.1)		√	[36]
**53**	16.5	Macrocyclic glycoterpenoid	Syphonoside [a]		831.3645		785<br>683<br>623<br>581<br>521<br>479<br>461<br>317		$C_{38}H_{58}O_{17}$ (0.1)	√	√	[37]

**54**	16.89	Flavone	6-Hydroxyl luteolin-*O*-malonyl glucoside [e]	549.0879	551.1052	505<br>463<br>301<br>271<br>255<br>179	303	$C_{24}H_{22}O_{15}$ (1.3)	√		[38]
**55**	17.33	Flavone	6-Hydroxyl luteolin [a]		303.0505			$C_{15}H_{10}O_{7}$ (-1.99)		√	[28]
**56**	17.33	Flavone	6-Hydroxyl luteolin -*O*-xyloside [e]	433.0776	435.0931	301	303	$C_{20}H_{18}O_{11}$ (2.1)		√	[39]
**57**	17.38	Cinnamic acid	*O*-Caffeoyl tartaric acid isomer [d]	311.0561		179<br>149<br>135<br>113		$C_{13}H_{12}O_{9}$ (1.0)		√	[13]
**58**	17.4	Cinnamic acid	Di-*O*-caffeoyl tartaric acid (Chicoric acid) [d]	473.0724		311<br>293<br>179<br>149<br>113		$C_{22}H_{18}O_{12}$ (0.5)		√	[15]
**59**	17.5	Flavone	Chrysoeriol 7-*O*-glucoside sulphate (Thalassiolin B) [c]	541.0635	543.0816	461<br>299	461<br>299	$C_{22}H_{22}O_{14}S$ (-3.6)		√	[40]
**60**	17.55	Flavone	Apigenin7-*O*-glucoside sulphate<br>(Thalassiolin C) [c]	511.0550		269		$C_{21}H_{20}O_{13}S$ (0.23)		√	[40]
**61**	17.77	Flavone	Scutellarein-*O*-glucoside[d]		449.1086		287<br>245<br>177	$C_{21}H_{20}O_{11}$ (-2.1)		√	[41]
**62**	17.78	Flavone	Monohydroxy trimethoxy flavone-*O*- glucoside [e]		491.1555		329<br>314	$C_{24}H_{26}O_{11}$ (1.48)	√		[42, 43]
**63**	17.9	Flavone	Apigenin 7-*O*-glucoside [b,c]	431.0988	433.1135	269	271	$C_{21}H_{20}O_{10}$ (1.32)	√	√	GNPS libraries, [44]
**64**	17.9	Cinnamic acid	Coumaric acid [d,c,d]	163.0402		119		$C_{9}H_{8}O_{3}$ (0.8)	-	√	[45]
**65**	18.0	Lignan	Trilobatin E [e]		889.1082		563	$C_{43}H_{36}O_{21}$ (0.14)	√	-	[46]

								455<br>301				
**66**	18.03	Flavone	Chrysoeriol-*O*-glucoside [b]	461.1095		463.1247	299<br>283	301	$C_{22}H_{22}O_{11}$ (0.9)	√	√	[44]
**67**	18.37	Flavone	Isoscutellarein 7-*O*-xyloside [c]	417.0828			285<br>255<br>227		$C_{20}H_{18}O_{10}$ (-0.4)		√	[47]
**68**	18.4	Flavone	Carboxymethyl-dihydroxyflavone [e]	311.0566			267<br>175		$C_{17}H_{12}O_{6}$ (0.6)		√	[48]
**69**	18.47	Flavone	Isoscutellarein 7-*O*-glucoside [c]	447.0931			285<br>227		$C_{21}H_{20}O_{11}$ (0.5)		√	[47]
**70**	18.5	Flavone	6-Hydroxy luteolin-*O*-acetyl glucoside [d, e]	505.0976		507.1147	301<br>300<br>271<br>255<br>179	303	$C_{23}H_{22}O_{13}$ (-2.7)	√		[28]
**71**	18.6	Chalcone	Phloridzin (Phloretin-*O*-glucoside) [d,e]	435.1296			273<br>179<br>167		$C_{21}H_{24}O_{10}$(0.1)		√	[39]
**72**	18.91	Steviol glycoside	Rebaudioside B [e]	803.3714			641<br>479<br>317<br>161		$C_{38}H_{60}O_{18}$ (0.9)	√		[49]
**73**	19.03	Macrocyclic glycoterpenoid	Syphonoside-*O*-acetate [b]		873.3759		827<br>665<br>623<br>521<br>461<br>317		$C_{40}H_{60}O_{18}$ (0.8)	√	√	[50]
**74**	19.25	Cinnamic acid	*O*-Caffeoyl-*O*-coumaroyl tartaric acid (Isomer I) [d]	457.0777			295<br>163<br>149		$C_{22}H_{18}O_{11}$ (0.1)		√	[15]
**75**	19.26	Flavone	Pedalitin-*O*-glucoside sulphate [f]	557.0601			315<br>300<br>241		$C_{22}H_{22}O_{15}S$ (1.3)		√	[51]

**76**	19.39	Flavone	Apigenin -*O*- malonyl glucoside [b]			519.1144		271	$C_{24}H_{22}O_{13}$ (2.1)	√		[52]
**77**	19.53	Acylglycerol	Dihydroxy linolenoyl glycerol di-*O*-hexoside (Isomer I) [e]		753.3548		707<br>415<br>397<br>291<br>235<br>179<br>119		$C_{33}H_{56}O_{16}$ (0.4)	√	√	[53]
**78**	19.59	Cinnamic acid	*O*-Caffeoyl-*O*-coumaroyl tartaric acid (Isomer II) [d]	457.0777			295<br>163<br>149		$C_{22}H_{18}O_{11}$ (0.1)		√	[15]
**79**	19.6	Flavone	Chrysoeriol -*O*- malonyl-glucoside [e]			549.1252		301	$C_{25}H_{24}O_{14}$ (2.3)	√		[54]
**80**	19.68	Cinnamic acid	*O*-Caffeoyl-*O*- feruloyl tartaric acid [e]	487.0880			325<br>193<br>179<br>161<br>149<br>135<br>113		$C_{23}H_{20}O_{12}$ (1.0)		√	[55]
**81**	19.7	Cinnamic acid	*O*-Feruloyl tartaric acid (fertaric acid) [e]	325.0565			193		$C_{14}H_{14}O_{9}$ (5.7)		√	[56]
**82**	19.9	Flavone	Dihydroxy dimethoxy flavone [d,e]	313.0718			175<br>147		$C_{17}H_{14}O_{6}$ (0.3)		√	[39]
**83**	20.22	Flavone	6-hydroxyl luteolin-*O*-coumaroyl glucoside (isomer I) [d]	609.1250			463<br>301		$C_{30}H_{26}O_{14}$ (0.0)		√	[28]
**84**	20.29	Flavone	Dihydroxydimethoxy flavone -*O*-glucoside [e]			477.1403		315	$C_{23}H_{24}O_{11}$ (2.7)	√	√	[27]
**85**	20.44	Flavone	Luteolin-*O*-sulphate [b, c, d]	364.9965			285		$C_{15}H_{10}O_{9}S$ (2.3)	√	√	[39]

**86**	20.78	Fatty acid	Trihydroxy octadecadienoic acid (Isomer I) [e]	329.2304			270<br>224		$C_{18}H_{32}O_5$ (5.59)		√	[53]
**87**	20.89	Flavone	6-hydroxyl luteolin -*O*-coumaroyl glucoside (isomer II) [d]	609.1247			463<br>301		$C_{30}H_{26}O_{14}$ (0.42)		√	[28]
**88**	21.17	Flavonoid<br>Flavone glycoside	Scutellarein -*O*- coumaroyl glucoside [d]	593.1300			285<br>257<br>243		$C_{30}H_{26}O_{13}$ (0.1)		√	[28]
**89**	21.24	Flavone	Apigenin-*O*-acetyl glucoside [e]	473.1088		475.1249	269	271	$C_{23}H_{22}O_{11}$ (1.0)	√		[44]
**90**	21.25	Acylglycerol	Dihydroxy linolenoyl glycerol-*O*-hexoside (Isomer I) [e]		591.3027	383<br>291<br>275<br>253			$C_{27}H_{46}O_{11}$ (0.8)	√	√	[53]
**91**	21.29	Cinnamic acid	Di-*O*-coumaroyl tartaric acid (Isomer I) [d]	441.0828			277<br>163		$C_{22}H_{18}O_{10}$ (0.2)		√	[15]
**92**	21.38	Benzoic acid	Ethyl protocatechuic acid [e]	181.0507			153<br>109<br>108		$C_9H_{10}O_4$ (0.2)		√	[57]
**93**	21.48	Cinnamic acid	Ferulic acid [c,d]	193.0505			161<br>135<br>134		$C_{10}H_{10}O_4$ (0.9)		√	[58]
**94**	21.68	Flavone	Chrysoeriol-*O*-glucoside [d]	461.1092		463.1247	299	301	$C_{22}H_{22}O_{11}$ (0.9)	√	√	[44]
**95**	21.7	Cinnamic acid	Di-*O*-coumaroyl tartaric acid acid(Isomer II) [d]	441.0824			277<br>163		$C_{22}H_{18}O_{10}$ (-0.1)		√	[15]
**96**	21.7	Steviol glycoside	Rebaudioside-*O*-acetate [f]	845.3822			683<br>521<br>317		$C_{40}H_{62}O_{19}$ (1.1)	√		

**97**	22.11	Flavone	Genkwanin 4′-*O*-glucoside[d]		491.1281	447.1295	445<br>283	285	$C_{22}H_{22}O_{10}$ (2.1)	√	√	[59]
**98**	22.26	Flavone	5,4′-Dihydroxy 6,7-dimethoxy flavone (Cirsimaritin) [e]	313.0719		315.0868	298<br>175<br>131		$C_{17}H_{14}O_6$ (0.2)	√	√	GNPS libraries
**99**	22.33	Flavone	5,4′-Dihydroxy 6,7-dimethoxy flavone -*O*-glucoside (Cirsimarin) [e]	475.1245		477.1399	313<br>298	315	$C_{23}H_{24}O_{11}$ (1.5)	√	√	GNPS libraries
**100**	22.57	Steviol glycoside	Rubusoside [f]	641.3191		-	479<br>317<br>161	-	$C_{32}H_{50}O_{13}$ (0.4)	√		[60]
**101**	22.9	Flavone	Scutellarein [a, d]	285.0405			257<br>243<br>199<br>170<br>151<br>133		$C_{15}H_{10}O_6$ (0.3)		√	[39]
**102**	24.38	Acylglycerol	Hydroxy linolenoyl glycerol-di-*O*-hexoside (Isomer I) [f]		737.3597	-	415<br>367<br>293<br>235<br>183	-	$C_{33}H_{56}O_{15}$ (0.6)	√	√	[61]
**103**	24.2	Cinnamaldehyde	Sinapaldehyde [e]	207.0663			179<br>161		$C_{11}H_{12}O_4$ (0.2)		√	[62]
**104**	24.80	Acylglycerol	Hydroxy linolenoyl glycerol -di-*O*-hexoside (Isomer II) [f]		737.3609	-	691<br>415<br>367<br>293<br>275<br>235<br>183	-	$C_{33}H_{56}O_{15}$ (0.4)	√	√	[61]

**105**	24.9	Flavone	Monohydroxytetramethoxy flavone [f]	-		359.1135	-	344<br>315	$C_{19}H_{18}O_7$ (2.8)	√		[63]
**106**	24.95	Glycerophospho choline lipid	Hydroxy linoleoylglycero-phosphocholine (Isomer I) [f]		578.3089		518<br>293<br>275<br>223<br>183		$C_{26}H_{48}NO_8P$ (0.3)	√	√	[61]
**107**	25.09	Acylglycerol	Hydroxy linolenoyl glycerol -di-*O*-hexoside (Isomer III) [f]		737.3603		415<br>367<br>293<br>275<br>235<br>179		$C_{33}H_{56}O_{15}$ (1.1)	√	√	[61]
**108**	25.38	Flavone	Monohydroxy trimethoxy flavone [c]			329.1029		314<br>286	$C_{18}H_{16}O_6$ (3.4)	√		[13]
**109**	25.6	Flavone	Apigenin [d]	269.0453		271.0605	227<br>151<br>117	153	$C_{15}H_{10}O_5$ (0.8)	√	√	[28]
**110**	25.7	Acylglycerol	Dihydroxy linolenoyl glycerol di-*O*-hexoside (Isomer II) [e]		753.3548		707<br>415<br>397<br>309<br>291<br>235<br>183<br>119		$C_{33}H_{56}O_{16}$ (0.4)	√	√	[53]
**111**	26.29	Flavone	Hispidulin [d,e]	299.0571			285<br>284<br>255		$C_{16}H_{11}O_6$ (0.6)		√	GNPS Library [28]
**112**	26.92	Glycerophospho choline lipid	Hydroxy-octadecadienoyl-glycerophosphocholine [e]		580.3258		520<br>295<br>224		$C_{26}H_{50}NO_8P$ (0.4)		√	[61]

							183					
**113**	27.51	Acylglycerol	Hydroxy linolenoyl glycerol-*O*-hexoside (Isomer I) [e]		575.3080		293<br>275<br>253<br>171		$C_{27}H_{46}O_{10}$ (0.0)	√	√	[64]
**114**	27.81	Acylglycerol	Hydroxy linolenoyl glycerol-*O*-hexoside (Isomer II) [e]	529.3013			293<br>275<br>253<br>171		$C_{27}H_{46}O_{10}$ (1.0)	√	√	[64]
**115**	27.89	Cinnamic acid	Coumaric acid ethyl ester [d]	191.0716			163<br>145<br>119		$C_{11}H_{12}O_3$ (1.0)	√	√	[65]
**116**	28.0	Fatty acid	Trihydoxy octadecenoic acid (Isomer II) [e]	329.2333			311<br>201		$C_{18}H_{34}O_5$ (0.0)		√	[53]
**117**	28.2	Coumarin	Dihydrodihydroxy-methyl-isocoumarin [e]			195.0657		167<br>152<br>124	$C_{10}H_{10}O_4$ (3.1)	√		[66]
**118**	28.32	Acylglycerol	Dihydroxy linolenoyl glycerol-*O*-hexoside (Isomer II) [e]	545.2972			293<br>291<br>275<br>253		$C_{27}H_{46}O_{11}$ (0.8)	√	√	[64]
**119**	28.49	Flavone	Chrysoeriol [b]	299.056			285<br>284<br>183		$C_{16}H_{12}O_6$ (0.16)	√	√	GNPS library [67]
**120**	28.95	Fatty acid	Dihydroxy-tetradecanoic acid [e]	259.1916			213<br>141		$C_{14}H_{28}O_4$ (-0.6)		√	[68]
**121**	31.03	Fatty acid	Trihydoxy octadecenoic acid (Isomer III) [e]	329.2332			311<br>199		$C_{18}H_{34}O_5$ (-0.1)		√	[53]

**122**	31.15	Acylglycerol	Octadecatrienoyl glycerol-*O*-dihexoside[e]	721.3655			415<br>397<br>277<br>235<br>179<br>119		$C_{34}H_{58}O_{16}$ (0.4)	√	√	GNPS library [69]
**123**	31.16	Acylglycerol	Linolenoyl-glycerol (Isomer I) [e]			353.2694		261	$C_{21}H_{36}O_4$ (2.4)	√		[70]
**124**	31.86	Flavone	Genkwanin (Apigenin 7-methyl ether) [b,d]	283.0608			269<br>268		$C_{16}H_{12}O_5$ (1.35)	√	√	GNPS library [50]
**125**	32.59	Flavone	Dihydroxy dimethoxy flavone	313.0719			298<br>283<br>269<br>255		$C_{17}H_{14}O_6$ (0.2)	√	√	[71]
**126**	32.9	Glycerophosphate lipid	Hydroxy-octadecatrienoyl glycerophosphate (Isomer I) [e]		475.2470		293<br>275<br>235<br>181<br>153<br>125		$C_{21}H_{35}O_7P$ (0.4)	√	√	[64]
**127**	33.6	Glycerophosphate lipid	Hydroxy-octadecatrienoyl glycerophosphate (Isomer II) [e]		475.2468		293<br>275<br>235<br>199<br>181<br>153<br>125		$C_{21}H_{35}O_7P$ (0.3)	√	√	[64]
**128**	33.75	Fatty acid	Palmitic-oleic dimer hexoside (Isomer I) [e]	699.3811			415<br>397<br>287<br>235<br>179<br>119		$C_{32}H_{60}O_{16}$ (0.7)	√	√	[69]

**129**	34.62	Glycerophospho choline lipid	Octadecadienoyl-sn-glycero-phosphocholine [e]		564.3308	520.337	504<br>279<br>242<br>224<br>183	184	$C_{26}H_{50}NO_7P$ (0.2)	√	√	GNPS library, [53]
**130**	34.72	Fatty acid	Palmitic-oleic dimer hexoside (Isomer II) [e]	699.3815			415<br>397<br>287<br>235<br>179<br>119		$C_{32}H_{60}O_{16}$ (0.9)	√	√	[69]
**131**	34.95	Glycerophospha te lipid	Linolenoyl glycerophosphate [e]		459.2519		277<br>181<br>153		$C_{21}H_{35}O_6P$ (0.4)	√	√	[64]
**132**	35.08	Glycerophospha te lipid	Nonadecatrienoyl-glycerophosphate [e]		491.2411		445<br>291<br>263<br>223<br>181<br>153		$C_{22}H_{39}O_7P$(0.4)	√	√	[64]
**133**	35.4	Acylglycerol	Linolenoyl-glycerol (Isomer II) [e]	-		353.2694	-	261	$C_{21}H_{36}O_4$ (2.6)	√		[70]
**134**	35.52	Acylglycerol	Octadecatrienoyl glycerol-*O*-hexoside [e]		559.3127		351<br>277<br>253		$C_{28}H_{48}O_{11}$(0.5)	√	√	GNPS library
**135**	36.06	Glycerophospha te lipid	Hydroxy-octadecadienoyl glycero-phosphate (Isomer I) [e]		477.2624		295<br>277<br>183<br>153		$C_{21}H_{37}O_7P$ (0.2)	√	√	[64]
**136**	36.44	Glycerophospho choline lipid	Hexadecanoyl-glycero-phosphocholine=Palmitoyl-glycerophosphocholine [e]		540.3306	496.3401	480<br>255<br>224<br>183	313<br>184	$C_{24}H_{50}NO_7P$ (0.2)	√	√	[53]

**137**	36.54	Fatty acid	Hydroxy-octadecatrienoic acid (Isomer I) [e]	293.2123		275<br>223<br>183<br>171<br>155	$C_{18}H_{30}O_3$ (0.1)	√	√	[72, 73]
**138**	36.68	Glycerophosphate lipid	Hydroxy-octadecadienoyl glycero-phosphate (Isomer II) [e]		477.2630	295<br>277<br>195<br>181<br>153	$C_{21}H_{37}O_7P$ (1.5)	√	√	[64]
**139**	36.74	Fatty acid	Hydroxy-octadecatrienoic acid (Isomer II) [e]	293.2143		275<br>223<br>183<br>171<br>155	$C_{18}H_{30}O_3$ (1.3)	√	√	[72]
**140**	37.05	Fatty acid	Hydroxy-octadecatrienoic acid (Isomer III) [e]	293.2119		275<br>223<br>205<br>195	$C_{18}H_{30}O_3$ (0.2)	√	√	[72]
**141**	37.46	Glycerophosphate lipid	Hydroxy-octadecadienoyl glycero-phosphate (Isomer III) [e]		477.2620	295<br>277<br>199<br>181<br>153	$C_{21}H_{37}O_7P$ (0.4)	√	√	[64]
**142**	38.29	Lysophosphatidylglycerols (Acyl-glycero-phosphoglycerol lipids)	Heptadecadienoyl-glycero-phosphoglycerol (Isomer I) [e]	493.2576		363<br>293<br>245<br>181<br>153	$C_{23}H_{43}O_9P$ (0.9)	√	√	[64]
**143**	38.91	Fatty acid	Hydroxyoctadecadienoic acid [e] (Dimorphecolic acid)	295.2280		279<br>277<br>259<br>195<br>183<br>171	$C_{18}H_{32}O_3$(-0.4)	√	√	GNPS library [53]

					155				
**144**	39.31	Lysophosphatidylglycerols (Acyl-glycero-phosphoglycerol lipids)	Heptadecadienoyl-glycero-phosphoglycerol (Isomer II) [e]	493.2576	293<br>245<br>181<br>153	$C_{23}H_{43}O_9P$ (0.9)	√	√	[64]

[a] Flavonoid aglycones isolated in the present study

[b] Compounds previously isolated from *Hs*

[c] Compounds previously isolated from *Th*

[d] Compounds previously reported in other seagrasses

[e] Compounds detected for the first time from the studied species

[f] Compounds not previously reported in nature

Table S3: Results of enzymes inhibition assays of *Hs* and *Th* extracts

	**α-amylase inhibition assay**		**β- glucosidase inhibition assay**		**pancreatic lipase inhibition assay**	
	% of inhibition at 600 µg/ml	IC_{50} (µg/ml)	% of inhibition at 300 µg/ml	IC_{50} (µg/ml)	% of inhibition at 100 µg/ml	IC_{50} (µg/ml)
Hs extract	87.5 ± 5.9	250.62 ± 8.2	48 ± 4.5	380 ± 3.5	94.7 ± 6.1	23.05 ± 3.5
Th extract	92.9± 6.8	230.28 ± 8.5	52.5±4.8	332.5±3.4	94.8 ± 5.4	23.7±3.2
Acarbose	91.1 ± 7.2	230.85 ± 2.45	62.1 ± 7.2	250.76 ± 1.23		28.96 ± 6.4
Orlistat					92 ± 6.4	28.96 ± 6.4

Table S4: Summary of the biomarkers in different study groups of diabetic rats

	**D**	**G**	***Th* 100**	***Th* 200**	***Hs* 100**	***Hs* 200**
**Glucose (mg/dl)**	379.8 ± 30.5	88.0 ± 6.4	196.7 ± 10.3[a,b,d,e]	181.7 ± 10.3[a,b,c,f]	119.3 ± 3.1[a,b,c,f]	101.0 ± 3.0[a,b,d,e]
**Insulin (mIU/ml)**	1.3 ± 0.2	5.2 ± 0.6	1.8 ± 0.1[a,b,e]	1.9 ± 0.2[a,b,f]	6.0 ± 0.2[a,b,c,f]	6.7 ± 0.3[a,b,d,e]
**GLUT2 (ng/g tissue)**	2.5 ± 0.4	26.4 ± 2.4	6.9 ± 0.8[a,b,d,e]	11.7 ± 0.9[a,b,c,f]	14.5 ± 1.0[a,b,c,f]	22.6 ± 1.0[a,b,d,e]
**NO (µmol/l)**	2.9 ± 0.2	51.0 ± 5.3	8.1 ± 0.5[a,b,d,e]	8.9 ± 0.4[a,b,c,f]	26.7 ± 2.2[a,b,c,f]	36.6 ± 2.3[a,b,d,e]
**MDA (ng/g tissue)**	25.2 ± 1.5	2.5 ± 0.3	12.3 ± 0.4[a,b,d,e]	9.8 ± 1.1[a,b,c,f]	4.0 ± 0.2[a,b,c,f]	3.4 ± 0.2[a,b,d,e]
**Total Cholesterol (mg/dl)**	125.8 ± 7.0	35.0 ± 1.8	83.0 ± 3.8[a,b,d,e]	72.8 ± 5.5[a,b,c,f]	39.2 ± 1.2[a,b,c,f]	37.3 ± 1.0[a,b,d,e]
**HDL-Cholesterol (mg/dl)**	19.5 ± 1.4	36.0 ± 0.9	22.8 ± 0.8[a,b,d,e]	23.3 ± 0.8[a,b,c,f]	31.2 ± 1.2[a,b,c,f]	33.0 ± 0.9[a,b,d,e]
**Triglycerides (mg/dl)**	137.7 ± 7.3	37.3 ± 1.9	89.3 ± 3.1[a,b,d,e]	85.7 ± 3.3[a,b,c,f]	45.5 ± 2.9[a,b,c,f]	39.0 ± 0.9[a,b,d,e]

D: Diabetic control, G: Glibenclamide 6.5 mg kg^{-1}/day, *T*h100: *T*h 100 mg kg^{-1}/day, *Th* 200: *Th* 200 mg kg^{-1}/day, *Hs* 100: *Hs*100 mg kg^{-1}/day, *Hs* 200: *Hs* 200 mg kg^{-1}day.
Results are expressed as mean ± SD

a: Significant difference from diabetic control, b: Significant difference from glibenclamide group, c: Significant difference from *Th* 100 mg kg^{-1} / day group, d: Significant difference from *Th* 200 mg kg^{-1} / day group, e: Significant difference from *Hs* 100 mg kg^{-1} / day, f: Significant difference from *Hs* 200 mg kg^{-1} / day.

Supplementary Figures

Fig. S1. HPLC profile of the *Halophila stipulaceae* (*Hs*) extract detected at wavelengths 210, 250, 285, 375 nm.

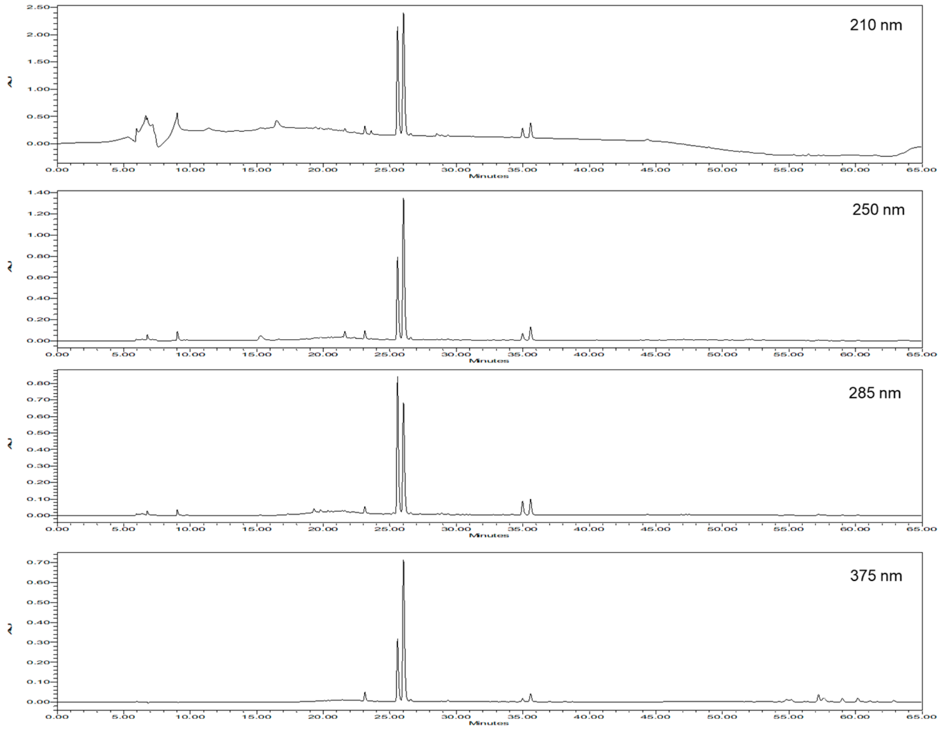

Fig. S2. HPLC profile of *Thalassia hempiricii* (*Th*) extract detected at wavelengths 210, 250, 285, 375 nm.

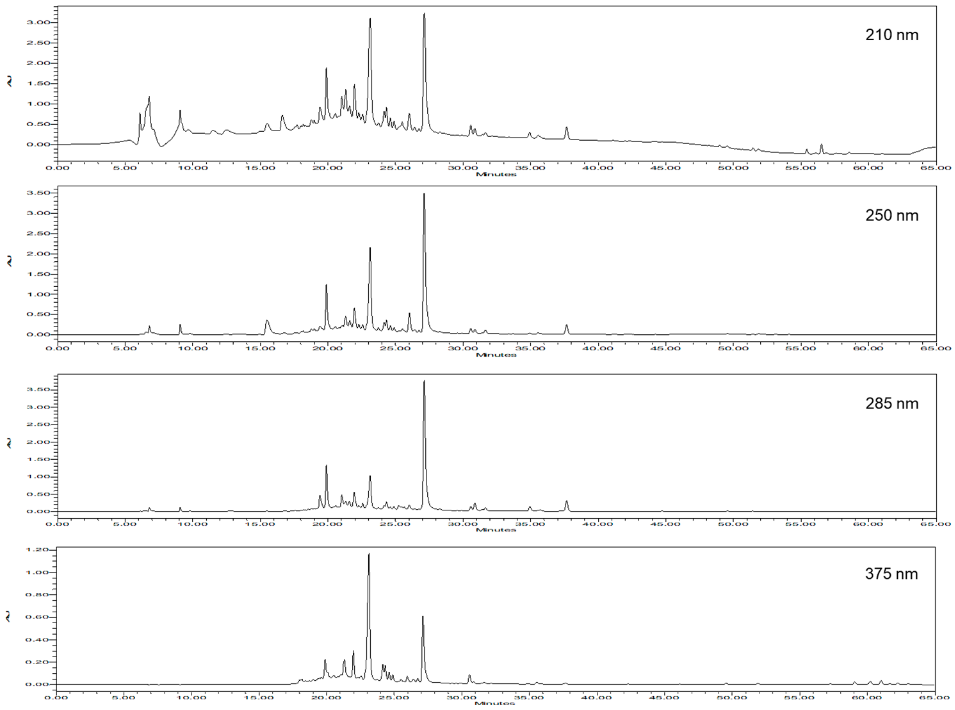

Fig. S3. The base peak chromatograms (BPC) of *Halophila stipulaceae* extract (blue) and *Thalassia hempiricii* extract (yellow) in the negative ionization mode.

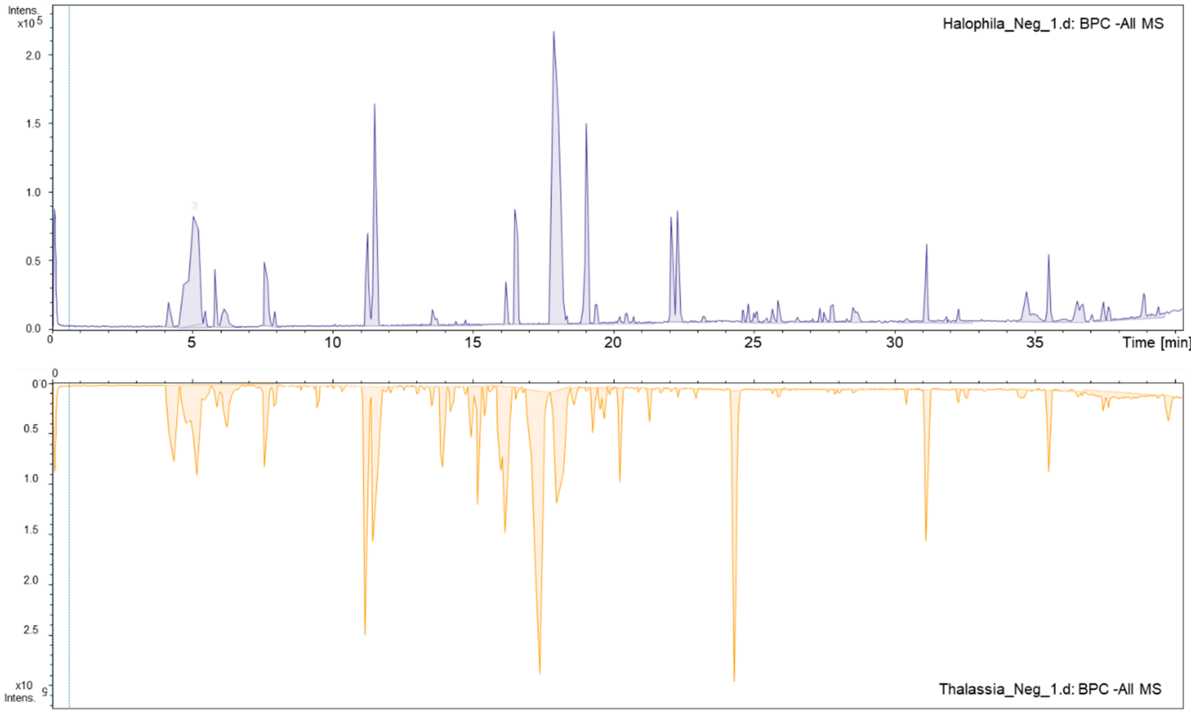

Fig. S4. The base peak chromatograms (BPC) of *Halophila stipulaceae* extract (blue) and *Thalassia hempiricii* extract (yellow) in the positive ionization mode.

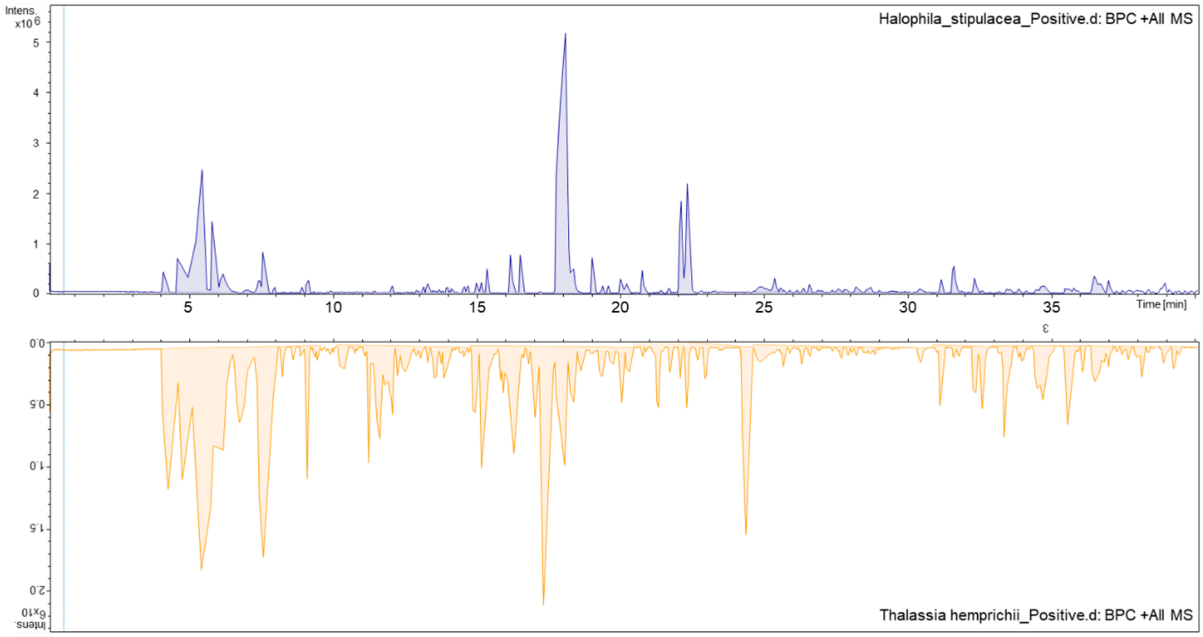

Fig. S5. Enlarged positive molecular network created using MS/MS data (positive mode) from *Halophila stipulaceae* (*Hs*) (purple nodes) and *Thalassia hempiricii* (*Th*) (yellow nodes). The network is displayed as a pie chart to reflect the relative abundance of each ion in both extracts. Black color corresponds to the solvent used as a blank.

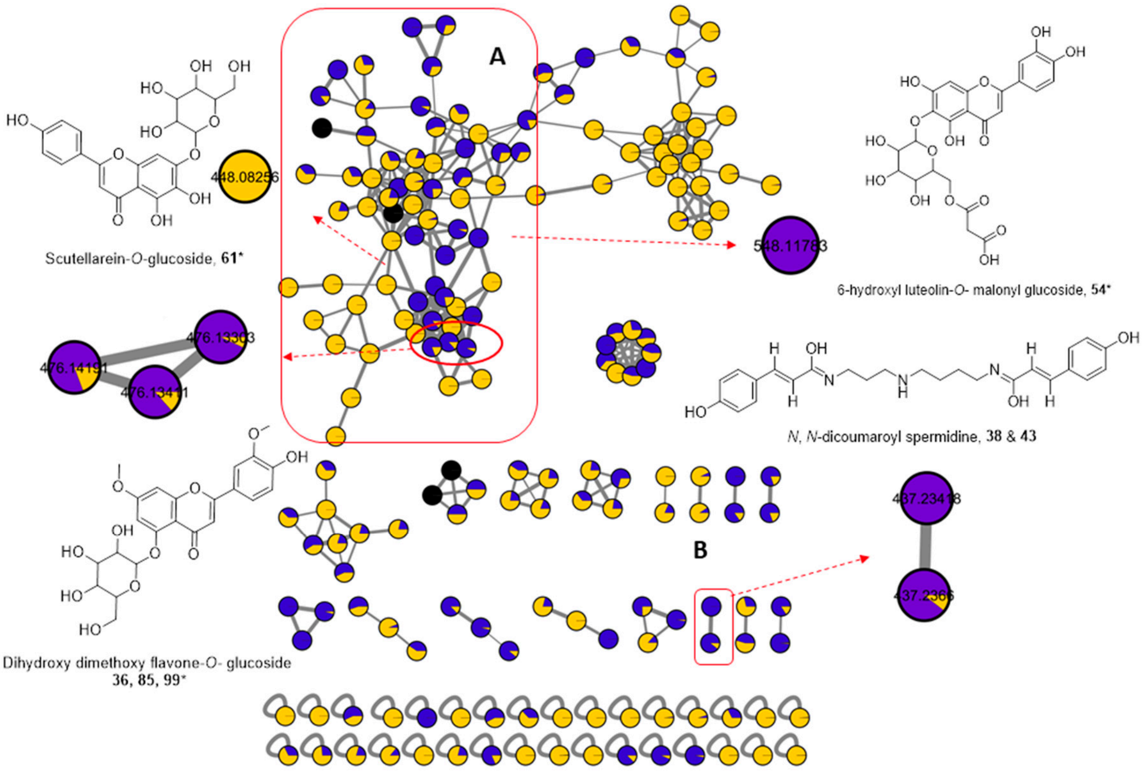

Fig. S6. Proposed fragmentation scheme and MS^2 spectrum of methoxy benzoic acid -*O*-sulphate, **16**.

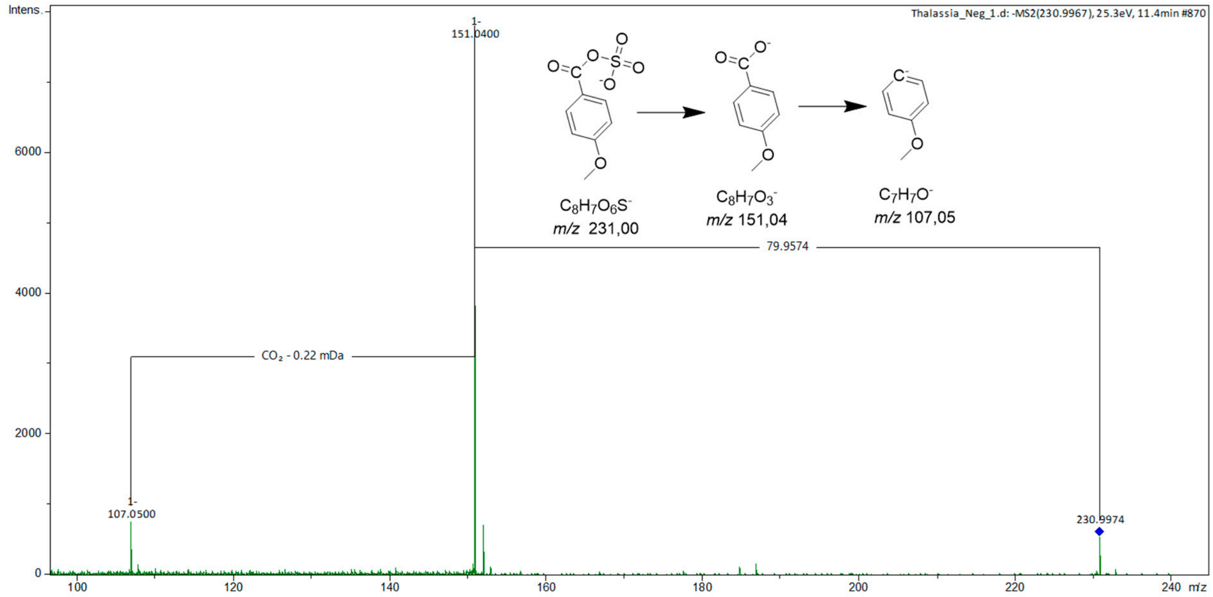

Fig. S7. Proposed fragmentation scheme and MS^2 spectrum of dimethoxy benzoic acid-*O*-sulphate, **30**.

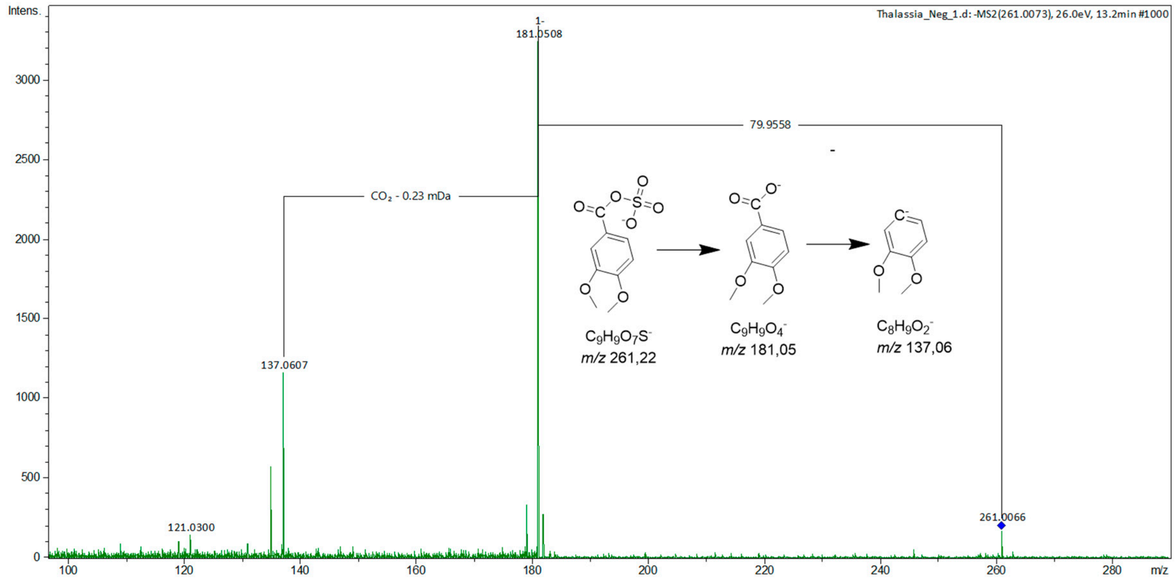

Fig. S8. Proposed fragmentation scheme and MS^2 spectrum of *O*-caffeoyl *O*-hydroxyldimethoxybenzoyl tartaric acid, **26.**

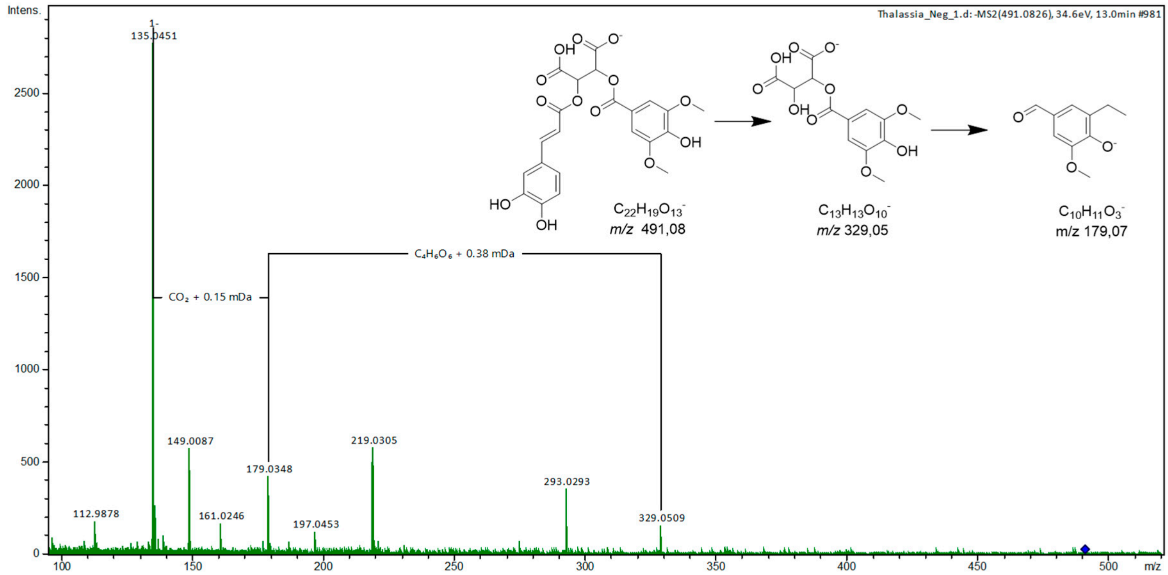

Fig. S9. Proposed fragmentation scheme and MS^2 spectrum of methoxypentahydroxyflavanone-*O*- hexoside**, 28.**

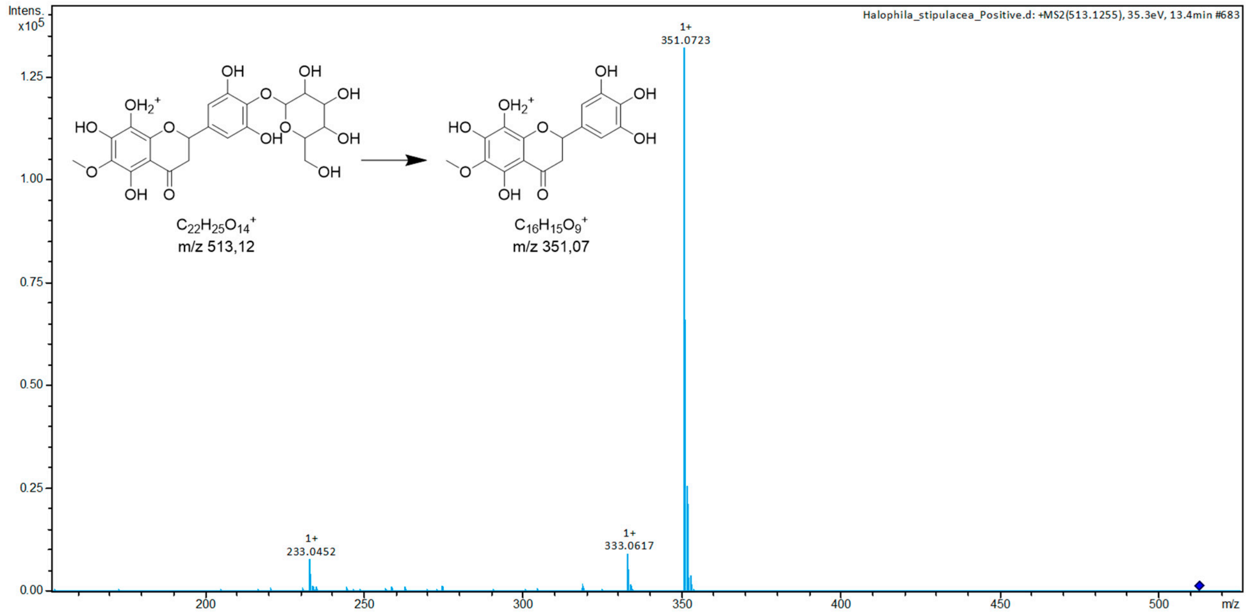

Fig. S10. Proposed fragmentation scheme and MS^2 spectra of acetylated rebaudioside, 96 (Upper) *vs.* its non-acetylated ascendant, rebaudioside**; 72** (Bottom)

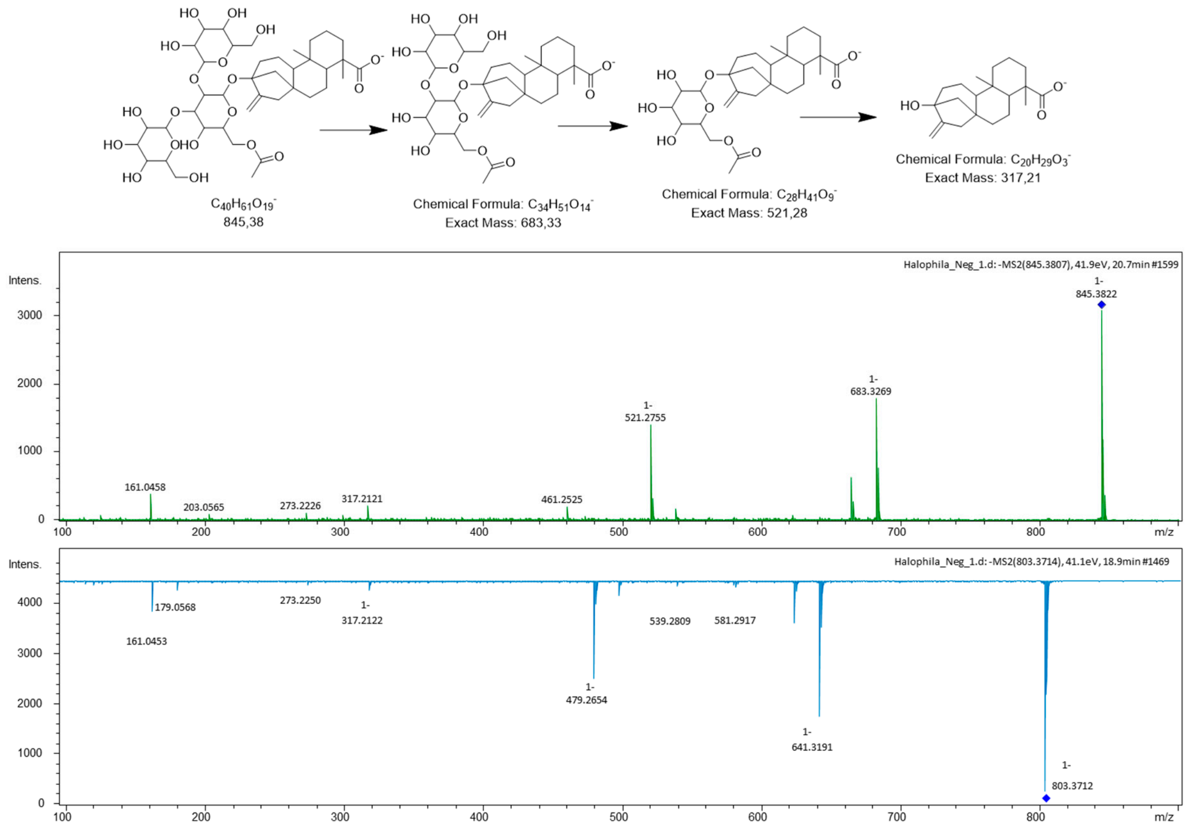

Fig. S11. EIC of syphonoside, **53** in both extracts (Upper: *Hs*; Bottom: *Th*)

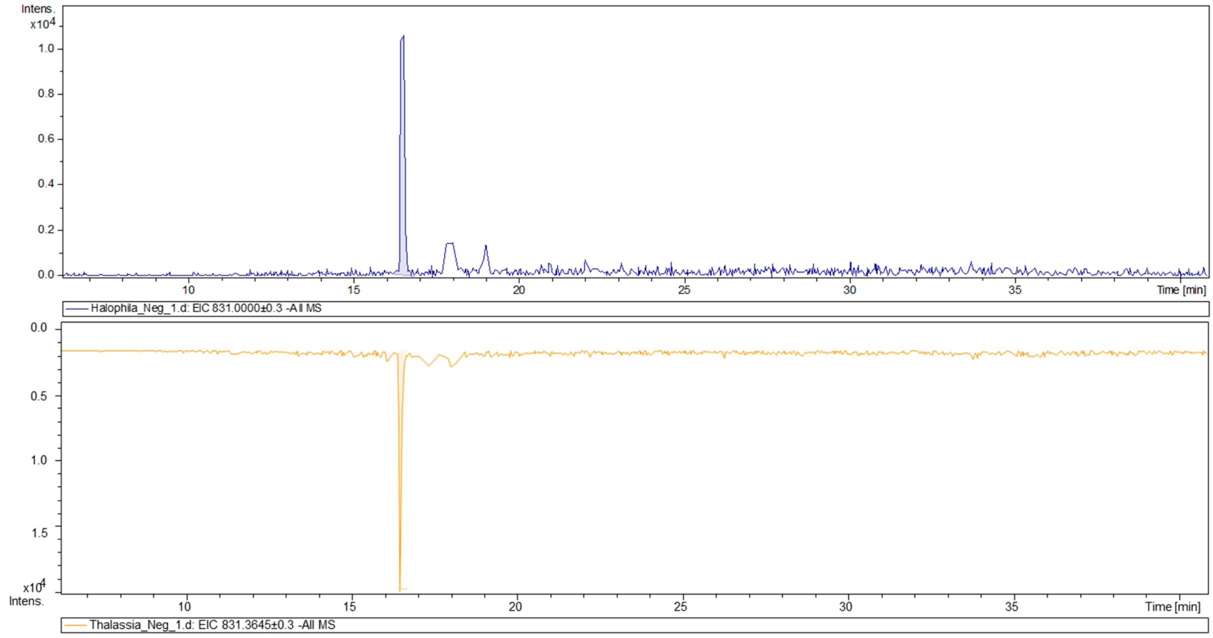

Fig. S12. Proposed fragmentation scheme and MS^2 spectrum of syphonoside, **53** in both extracts

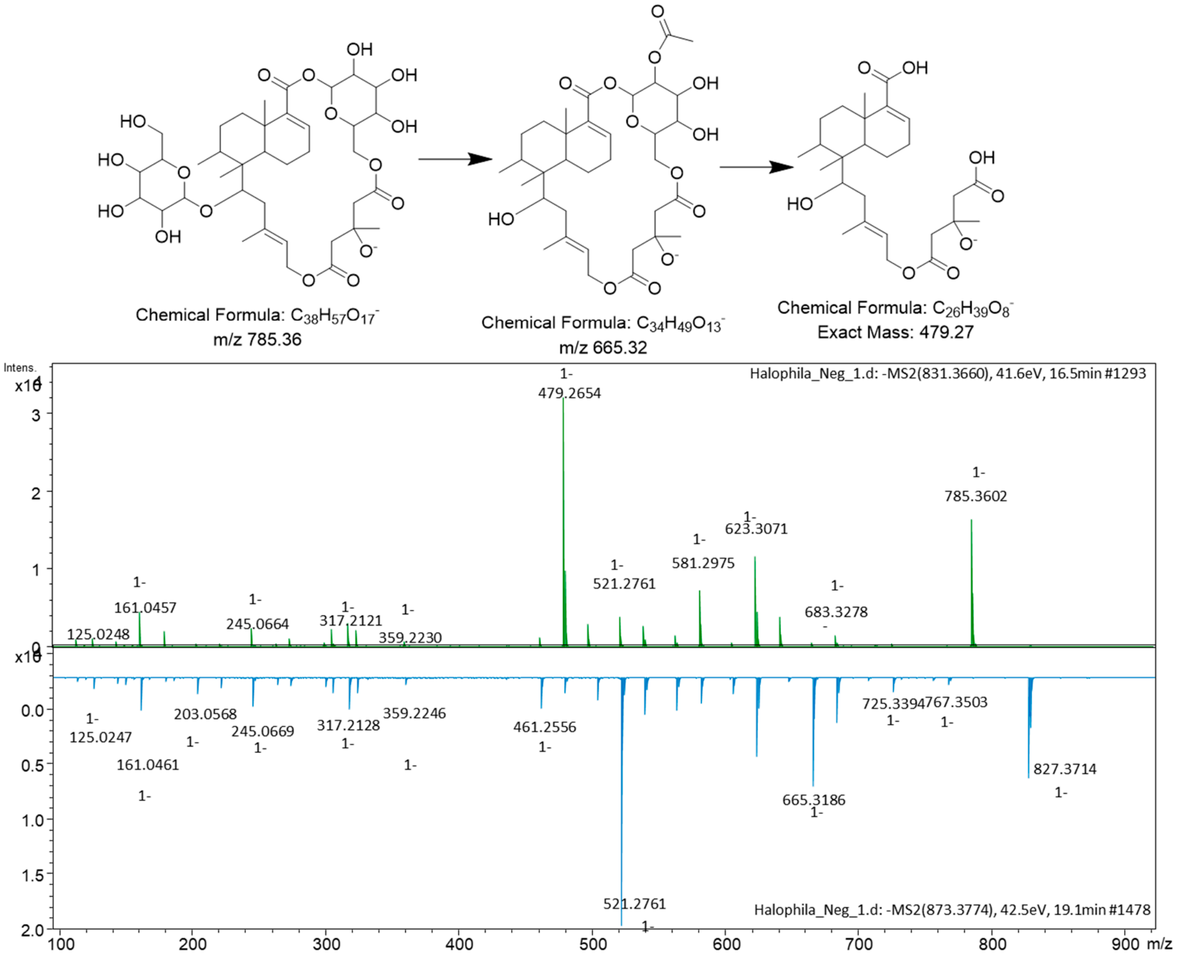

Results and Discussion

Compounds annotation

Lipids

Despite an early comparative study of 7 seagrasses (including *Th* and other *Halophila* species) described the occurrence of 30 saturated and unsaturated fatty acids with C_{12}-C_{30} chain length [74], no detailed reports ever since discussed the lipid profile of these two marine seagrasses. Comprehensively, cluster **A** in the negative MN (**Fig. 1**) unearthed a fertile lipid family with an array of different classes not previously described in these two genera.

Fatty acids

Eleven fatty acids were deconvoluted according to their molecular formula, fragmentation pattern, and the literature data reported by [53, 61, 69]. The negative MN *via* the GNPS2 platform allowed for the discrimination of the isomeric fatty acids besides proposing some hits from NIST as a rich lipid spectral library which were manually verified (Supplementary Table S2). For instance, two glycosylated fatty acid dimers, seen at Rt 33.75 & 34.72 min, were characterized as isomers of palmitic-oleic dimer hexoside; **128** & **130** (*m/z* 699.3811 $[M-H]^-$, $C_{32}H_{60}O_{16}$). Not only were glycosylated fatty acid deciphered but also hydroxylated fatty acids including dihydroxy-tetradecanoic acids; **120** (*m/z* 259.1916 $[M-H]^-$, $C_{14}H_{28}O_4$), isomers of trihydroxy octadecenoic acid; **87**, **117** & **122** (*m/z* 721.3655 $[M-H]^-$, $C_{34}H_{58}O_{16}$), and hydroxy-octadecatrienoic acid; **137**, **139** & **140** (*m/z* 293.2123 $[M-H]^-$, $C_{18}H_{30}O_3$) were tethered. Moreover, aminooctanedioic acid; **7** (*m/z* 188.0930 $[M-H]^-$, $C_8H_{15}NO_4$) was also characterized.

3.3.3.2. Acylglycerols (Glycerides)

Acylglycerols shaped the second class belonging to the lipids family, where the fatty acid moiety is esterified with glycerol through one of its hydroxyl groups. Thirteen acylglycerols were detected in the present study chiefly as mono-glycerides (Supplementary Table S2). Annotated mono-glycerides included isomers of linolenoyl-glycerol; **123** & **133**(*m/z* 353.2694 $[M+H]^+$, $C_{21}H_{36}O_4$). While mono glycosylated acylglycerols were detected at **90** (*m/z* 591.3027 $[M+CH_2O_2-H]^-$, $C_{27}H_{46}O_{11}$) & **118** (*m/z* 545.2972 $[M-H]^-$, $C_{27}H_{46}O_{11}$), annotated as dihydroxy linolenoyl glycerol *O*-hexoside. Besides, hydroxy linolenoyl glycerol *O*- hexoside at **113** & **114** (*m/z* 575.3080 $[M +CH_2O_2-H]^-$, $C_{27}H_{46}O_{10}$), and octadecatrienoyl glycerol *O*- hexoside **134** (*m/z* 559.3127 $[M +CH_2O_2-H]^-$, $C_{28}H_{48}O_{11}$).

Likewise, acylglycerols di-*O*- hexosides were described as dihydroxy linolenoyl glycerol di-*O*-hexoside; **77** & **110** (*m/z* 753.3548 $[M +CH_2O_2-H]^-$, $C_{33}H_{56}O_{16}$), hydroxy linolenoyl glycerol di-*O*- hexoside; **102**, **104** & **107** (*m/z* 737.3597 $[M +CH_2O_2-H]^-$, $C_{33}H_{56}O_{15}$), and

octadecatrienoyl glycerol di-*O*- hexoside; **122** (*m/z* 721.3655 [M -H]$^-$, $C_{34}H_{58}O_{16}$). Monogalactosyldiacylglyceride and digalactosyl- diacylglycerol were noticed previously in the seagrasses of Japan [75].

Phospholipids

Within the same lipidomic space, phosphorylated lipids were also recognized in the form of phosphocholines, glycerophosphates, and lysophosphatidylglycerols agreeing with [75] who highlighted the presence of such chemistries in the seagrasses. Interestingly, phospholipids were recounted to play a role in thermo-adpatation of the marine seagrass, *Zostera marina* [76].

Phosphocholines

Phosphocholines are phospholipids esterified with a choline moiety and are the structural elements of the biological membranes. The detected phosphocholine lipids were characterized by the molecular formula of NO_7P, and NO_8P heteroatom composition dependable of a phospholipid structure in which only one of the sn-1/sn-2 positions of the glycerol moiety is acylated with fatty acid and the nitrogenated head group corresponds to choline [53].

Detected phosphocholines were marked by their molecular anions [M+CH_2O_2-H]$^-$ as formic acid adducts aligning with the previous findings by [64], and yielding a main product ion [M-15]$^-$, conferring to the characteristic performance of phosphocholine lipid. Additionally, the detection of a product ion at *m/z* 183 correlates to the phosphocholine unit, which suggests these compounds to be lyso-phosphatidylcholine (Supplementary Table S2). Accordingly, detected phospholipids were annotated as octadecadienoyl-sn-glycerophosphocholine; **129** (*m/z* 564.3308 [M+CH_2O_2-H]$^-$, $C_{26}H_{50}NO_7P$), hydroxylinoleoyl glycerophosphocholine; **106** (*m/z* 578.3089 [M+CH_2O_2-H]$^-$, $C_{26}H_{48}NO_8P$), hydroxyl octadecadienoyl

glycerophosphocholine; **112** (*m/z* 580.3258 $[M+CH_2O_2-H]^-$, $C_{26}H_{50}NO_8P$), and hexadecanoyl glycerophosphocholine; **136** (*m/z* 540.3306 $[M+CH_2O_2-H]^-$, $C_{24}H_{50}NO_7P$).

Glycerophosphate lipids

Furthermore, another phospholipid class was encountered as glycerophosphate lipids which were distinguished by the neural loss of a dehydrated phosphoglycerol unit (-136 Da), and supported by the presence of the product ion at *m/z* 153 (Supplementary Table S2). This to include, isomers of hydroxy-octadecatrienoyl glycerophosphate; **126** & **127** (*m/z* 475.2470 $[M+CH_2O_2-H]^-$, $C_{21}H_{35}O_7P$), and hydroxyoctadecadienoyl glycerophosphate; (*m/z* 477.2624 $[M+CH_2O_2-H]^-$, $C_{21}H_{37}O_7P$) **136**, **138** & **141**. Analogously, linolenoyl and nonadecatrienoyl derivatives of glycerophosphate were detected at **133** (*m/z* 459.2519 $[M+CH_2O_2-H]^-$, $C_{21}H_{35}O_6P$) & **132** (*m/z* 491.2411$[M+CH_2O_2-H]^-$, $C_{22}H_{39}O_7P$), correspondingly.

Lysophosphatidylglycerols (Acyl-glycero-phosphoglycerol lipids)

In addition to the phospholipids and acylphospholipids, lysophosphatidylglycerols were the last defined class of lipids as glycero-phosphoglycerol in which the glycerol moiety is attached to a phosphate group linked to a fatty acid acylglycerol. The detected lysophosphatidylglycerols were exemplified by 2 isomers of heptadecadienoyl–glycerophosphoglycerol; **142** & **144** (*m/z* 493.2576 $[M -H]^-$, $C_{23}H_{43}O_9P$) as confirmed by product ions at *m/z* 153 and 245 corresponding to mono-dehydrated glycero-phosphate and glycerophosphoglycerol, respectively (Supplementary Table S2).

3.3.6. *Miscellaneous*

Two coumarins were assigned as dihydrodihydroxy-methyl-isocoumarin; **41** (*m/z* 177.0193 $[M-H]^-$, $C_9H_6O_4$) in *Th* and esculetin (6,7-dihydroxycoumarin); **117** (*m/z* 195.0657 $[M+H]^+$, $C_{10}H_{10}O_4$) in *Hs*. Differently, a characteristic lignan was attained in ***Hs*** as a scattered node, **65** (*m/z* 889.1082 $[M+H]^+$, $C_{43}H_{36}O_{21}$) with its MS^2 agreeing with that of trilobatin E [46].

References

1. Marzouk, M.M., et al., *Phenolics from Tanacetum sinaicum (Fresen.) Delile ex Bremer & Humphries (Asteraceae).* Biochemical Systematics and Ecology, 2016. **65**: p. 143-146.
2. Kassem, M.E., et al., *A sulphated flavone glycoside from Livistona australis and its antioxidant and cytotoxic activity.* Natural product research, 2012. **26**(15): p. 1381-1387.
3. Farid, M.M., et al., *Isoscutellarein 8, 4'-Dimethyl ether glycosides as cytotoxic agents and chemotaxonomic markers in Kickxia aegyptiaca.* Biocatalysis and Agricultural Biotechnology, 2019. **22**: p. 101431.
4. Abdalla, M.F., et al., *Flavone glycosides of Salvia triloba.* Phytochemistry, 1983. **22**(9): p. 2057-2060.
5. Saleh, N.A., et al., *Flavonoid glycosides of Artemisia monosperma and A. herba-alba.* Phytochemistry, 1985. **24**(1): p. 201-203.
6. Kelebek, H., et al., *HPLC determination of organic acids, sugars, phenolic compositions and antioxidant capacity of orange juice and orange wine made from a Turkish cv. Kozan.* Microchemical Journal, 2009. **91**(2): p. 187-192.
7. Taamalli, A., et al., *LC‐MS‐based metabolite profiling of methanolic extracts from the medicinal and aromatic species Mentha pulegium and Origanum majorana.* Phytochemical analysis, 2015. **26**(5): p. 320-330.
8. Kim, K.-J. and S.-K. Park, *Changes in major chemical constituents of green coffee beans during the roasting.* Korean Journal of Food Science and Technology, 2006. **38**(2): p. 153-158.
9. Janevska, S. and B. Tudzynski, *Secondary metabolism in Fusarium fujikuroi: strategies to unravel the function of biosynthetic pathways.* Applied microbiology and biotechnology, 2018. **102**(2): p. 615-630.
10. Peng, H., et al., *Major chemical constituents and antioxidant activities of different extracts from the peduncles of Hovenia acerba Lindl.* International journal of food properties, 2018. **21**(1): p. 2135-2155.
11. Kundu, A., *Vanillin biosynthetic pathways in plants.* Planta, 2017. **245**(6): p. 1069-1078.
12. Liu, M., et al., *Rapid screening of transferrin-binders in the flowers of Bauhinia blakeana Dunn by on-line high-performance liquid chromatography–diode-array detector–electrospray ionization–ion-trap–time-of-flight–mass spectrometry–transferrin–fluorescence detection system.* Journal of Chromatography A, 2016. **1450**: p. 17-28.
13. Qi, S., et al., *Phytochemical and chemotaxonomic investigation of seagrass Thalassia hemprichii (Ehrenb.) Aschers (Hydrocharitaceae).* Biochemical Systematics and Ecology, 2012. **43**: p. 128-131.
14. Ishii, T., et al., *Tichocarpols A and B, Two Novel Phenylpropanoids with Feeding-Deterrent Activity from the Red Alga Tichocarpus c rinitus.* Journal of natural products, 2004. **67**(10): p. 1764-1766.
15. Schütz, K., et al., *Characterization of phenolic acids and flavonoids in dandelion (Taraxacum officinale WEB. ex WIGG.) root and herb by high‐performance liquid chromatography/electrospray ionization mass spectrometry.* Rapid Communications in Mass Spectrometry: An International Journal Devoted to the Rapid Dissemination of Up‐to‐the‐Minute Research in Mass Spectrometry, 2005. **19**(2): p. 179-186.
16. Xie, J., et al., *Rapid identification and determination of 11 polyphenols in Herba lycopi by HPLC–MS/MS with multiple reactions monitoring mode (MRM).* Journal of Food Composition and Analysis, 2011. **24**(7): p. 1069-1072.
17. Neugart, S., S. Rohn, and M. Schreiner, *Identification of complex, naturally occurring flavonoid glycosides in Vicia faba and Pisum sativum leaves by HPLC-DAD-ESI-MSn and*

the genotypic effect on their flavonoid profile. Food Research International, 2015. **76**: p. 114-121.

18. Yang, L., et al., *High-performance liquid chromatography-diode array detection/electrospray ionization mass spectrometry for the simultaneous analysis of cis-, trans-and dihydro-2-glucosyloxycinnamic acid derivatives from Dendrobium medicinal plants.* Rapid Communications in Mass Spectrometry: An International Journal Devoted to the Rapid Dissemination of Up-to-the-Minute Research in Mass Spectrometry, 2007. **21**(12): p. 1833-1840.
19. Dias Silva, M.J., et al., *Bioassay-Guided Isolation of Fungistatic Compounds from Mimosa caesalpiniifolia Leaves.* Journal of natural products, 2019. **82**(6): p. 1496-1502.
20. Farag, M.A., et al., *Metabolite profiling in 18 Saudi date palm fruit cultivars and their antioxidant potential via UPLC-qTOF-MS and multivariate data analyses.* Food & function, 2016. **7**(2): p. 1077-1086.
21. Bagri, P., et al., *New flavonoids from Punica granatum flowers.* Chemistry of natural compounds, 2010. **46**(2): p. 201-204.
22. Aguado, M.I., et al., *Antioxidant and antibacterial activities of hydroalcoholic extracts from Aloysia polystachya griseb moldenke and Lippia turbinata griseb (verbenaceae).* Int J Pharm Pharm Sci, 2016. **8**(3): p. 393-395.
23. El-Ghffar, E.A.A., et al., *HPLC-ESI-MS/MS analysis of beet (Beta vulgaris) leaves and its beneficial properties in type 1 diabetic rats.* Biomedicine & Pharmacotherapy, 2019. **120**: p. 109541.
24. Hvattum, E. and D. Ekeberg, *Study of the collision-induced radical cleavage of flavonoid glycosides using negative electrospray ionization tandem quadrupole mass spectrometry.* Journal of Mass Spectrometry, 2003. **38**(1): p. 43-49.
25. Neveu, V., et al., *Phenol-Explorer: an online comprehensive database on polyphenol contents in foods.* Database, 2010. **2010**.
26. Gruz, J., O. Novák, and M. Strnad, *Rapid analysis of phenolic acids in beverages by UPLC–MS/MS.* Food chemistry, 2008. **111**(3): p. 789-794.
27. Švarc-Gajić, J., et al., *Characterisation of ginger extracts obtained by subcritical water.* The Journal of Supercritical Fluids, 2017. **123**: p. 92-100.
28. Meng, Y., et al., *Flavones and flavone glycosides from Halophila johnsonii.* Phytochemistry, 2008. **69**(14): p. 2603-2608.
29. Misra, B.B. and S. Dey, *Accumulation patterns of phenylpropanoids and enzymes in East Indian sandalwood tree undergoing developmental progression'in vitro'.* Australian Journal of Crop Science, 2013. **7**(5): p. 681.
30. Papetti, A., et al., *Polyphenolic profile of green/red spotted Italian Cichorium intybus salads by RP-HPLC-PDA-ESI-MSn.* Journal of Food Composition and Analysis, 2017. **63**: p. 189-197.
31. Mulabagal, V. and A.I. Calderón, *Liquid chromatography/mass spectrometry based fingerprinting analysis and mass profiling of Euterpe oleracea (açaí) dietary supplement raw materials.* Food chemistry, 2012. **134**(2): p. 1156-1164.
32. Tian, J., et al., *Hepatoprotective phenolic glycosides from Gymnema tingens.* Planta medica, 2013. **79**(09): p. 761-767.
33. Hawas, U.W. and L.T. Abou El-Kassem, *Thalassiolin D: a new flavone O-glucoside Sulphate from the seagrass Thalassia hemprichii.* Natural product research, 2017. **31**(20): p. 2369-2374.
34. Ferreres, F., et al., *HPLC-DAD-MS/MS-ESI screening of phenolic compounds in Pieris brassicae L. reared on Brassica rapa var. rapa L.* Journal of agricultural and food chemistry, 2008. **56**(3): p. 844-853.
35. Fuji, Y., et al., *Chemical characterization and biological activity in young sesame leaves (Sesamum indicum L.) and changes in iridoid and polyphenol content at different growth stages.* PloS one, 2018. **13**(3).
36. Hofmann, T., E. Nebehaj, and L. Albert, *The high-performance liquid chromatography/multistage electrospray mass spectrometric investigation and extraction*

optimization of beech (Fagus sylvatica L.) bark polyphenols. Journal of Chromatography a, 2015. **1393**: p. 96-105.
37. Gavagnin, M., et al., *Structure and absolute stereochemistry of syphonoside, a unique macrocyclic glycoterpenoid from marine organisms.* The Journal of organic chemistry, 2007. **72**(15): p. 5625-5630.
38. Ghouti, D., et al., *Phenolic profile and in vitro bioactive potential of Saharan Juniperus phoenicea L. and Cotula cinerea (Del) growing in Algeria.* Food & function, 2018. **9**(9): p. 4664-4672.
39. Zidorn, C., *Secondary metabolites of seagrasses (Alismatales and Potamogetonales; Alismatidae): Chemical diversity, bioactivity, and ecological function.* Phytochemistry, 2016. **124**: p. 5-28.
40. Rowley, D.C., et al., *Thalassiolins A–C: new marine-derived inhibitors of HIV cDNA integrase.* Bioorganic & medicinal chemistry, 2002. **10**(11): p. 3619-3625.
41. Sun, F., L. Shen, and Z. Ma, *Screening for ligands of human aromatase from mulberry (Mori alba L.) leaf by using high-performance liquid chromatography/tandem mass spectrometry.* Food chemistry, 2011. **126**(3): p. 1337-1343.
42. Dong, C., et al., *Flavanoids from the stems of Aquilaria sinensis.* Chinese journal of natural medicines, 2012. **10**(4): p. 287-291.
43. Boukhris, M.A., et al., *A dereplication strategy for the identification of new phenolic compounds from Anvillea radiata (Coss. & Durieu).* Comptes Rendus Chimie, 2016. **19**(9): p. 1124-1132.
44. Bitam, F., et al., *Chemical analysis of flavonoid constituents of the seagrass Halophila stipulacea: first finding of malonylated derivatives in marine phanerogams.* Biochemical Systematics and Ecology, 2010. **38**(4): p. 686-690.
45. Barros, L., et al., *Phenolic acids determination by HPLC–DAD–ESI/MS in sixteen different Portuguese wild mushrooms species.* Food and Chemical Toxicology, 2009. **47**(6): p. 1076-1079.
46. Scher, J.M., J. Zapp, and H. Becker, *Lignan derivatives from the liverwort Bazzania trilobata.* Phytochemistry, 2003. **62**(5): p. 769-777.
47. Hawas, U.W., *A new 8-hydroxy flavone O-xyloside sulfate and antibacterial activity from the Egyptian seagrass Thalassia hemprichii.* Chemistry of natural compounds, 2014. **50**(4): p. 629-632.
48. Cao, Y., et al., *Bioactive flavones and biflavones from Selaginella moellendorffii Hieron.* Fitoterapia, 2010. **81**(4): p. 253-258.
49. Pól, J., B. Hohnová, and T. Hyötyläinen, *Characterisation of Stevia rebaudiana by comprehensive two-dimensional liquid chromatography time-of-flight mass spectrometry.* Journal of Chromatography A, 2007. **1150**(1-2): p. 85-92.
50. Mollo, E., et al., *Factors promoting marine invasions: a chemoecological approach.* Proceedings of the National Academy of Sciences, 2008. **105**(12): p. 4582-4586.
51. Yagi, A., et al., *Antioxidative sulphated flavonoids in leaves of Polygonum hydropiper.* Phytochemistry, 1994. **35**(4): p. 885-887.
52. Han, Y., et al., *Comparative evaluation of different cultivars of Flos Chrysanthemi by an anti-inflammatory-based NF-κB reporter gene assay coupled to UPLC-Q/TOF MS with PCA and ANN.* Journal of ethnopharmacology, 2015. **174**: p. 387-395.
53. Napolitano, A., et al., *Multi-class polar lipid profiling in fresh and roasted hazelnut (Corylus avellana cultivar "Tonda di Giffoni") by LC-ESI/LTQOrbitrap/MS/MSn.* Food chemistry, 2018. **269**: p. 125-135.
54. Enerstvedt, K.H., M. Jordheim, and Ø.M. Andersen, *Isolation and identification of flavonoids found in Zostera marina collected in Norwegian coastal waters.* Analysis of polyphenolic content in marine and aquatic angiosperms from Norwegian coastal waters, 2016.
55. Carazzone, C., et al., *Identification of phenolic constituents in red chicory salads (Cichorium intybus) by high-performance liquid chromatography with diode array detection and electrospray ionisation tandem mass spectrometry.* Food Chemistry, 2013. **138**(2-3): p. 1062-1071.

56. Khoza, B., et al., *Identification of hydroxylcinnamoyl tartaric acid esters in Bidens pilosa by UPLC-tandem mass spectrometry.* South African Journal of Botany, 2016. **103**: p. 95-100.
57. Ma, Y., et al., *Separation and characterization of soluble esterified and glycoside-bound phenolic compounds in dry-blanched peanut skins by liquid chromatography–electrospray ionization mass spectrometry.* Journal of agricultural and food chemistry, 2014. **62**(47): p. 11488-11504.
58. Mohammed, M.M., et al., *Anti-influenza A virus activity of a new dihydrochalcone diglycoside isolated from the Egyptian seagrass Thalassodendron ciliatum (Forsk.) den Hartog.* Natural product research, 2014. **28**(6): p. 377-382.
59. Gong, J., et al., *Simultaneous qualitative and quantitative determination of phenylethanoid glycosides and flavanoid compounds in Callicarpa kwangtungensis Chun by HPLC-ESI-IT-TOF-MS/MS coupled with HPLC-DAD.* Analytical Methods, 2016. **8**(33): p. 6323-6336.
60. Molina-Calle, M., et al., *Development and application of a quantitative method based on LC–QqQ MS/MS for determination of steviol glycosides in Stevia leaves.* Talanta, 2016. **154**: p. 263-269.
61. Klockmann, S., et al., *Food fingerprinting: metabolomic approaches for geographical origin discrimination of hazelnuts (Corylus avellana) by UPLC-QTOF-MS.* Journal of agricultural and food chemistry, 2016. **64**(48): p. 9253-9262.
62. Seraglio, S.K.T., et al., *Development and validation of a LC-ESI-MS/MS method for the determination of phenolic compounds in honeydew honeys with the diluted-and-shoot approach.* Food Research International, 2016. **87**: p. 60-67.
63. Zheng, Y.y., et al., *Characterisation and classification of Citri Reticulatae Pericarpium varieties based on UHPLC-Q-TOF-MS/MS combined with multivariate statistical analyses.* Phytochemical Analysis, 2019. **30**(3): p. 278-291.
64. Pulfer, M. and R.C. Murphy, *Electrospray mass spectrometry of phospholipids.* Mass spectrometry reviews, 2003. **22**(5): p. 332-364.
65. Kannan, R.R.R., et al., *Phytochemical constituents, antioxidant properties and p-coumaric acid analysis in some seagrasses.* Food research international, 2013. **54**(1): p. 1229-1236.
66. Makhloufi, E., et al., *Chemical constituents of the extract Algerian Reutera lutea (Desf.) Maire,(Apiaceae).* Pharmacognosy Communications, 2013. **3**(2): p. 41.
67. Guan, Z., et al., *Identification and Quantitation of Phenolic Compounds from the Seed and Pomace of Perilla frutescens Using HPLC/PDA and HPLC–ESI/QTOF/MS/MS.* Phytochemical analysis, 2014. **25**(6): p. 508-513.
68. Saika, A., et al., *Screening and isolation of the liamocin-producing yeast Aureobasidium melanogenum using xylose as the sole carbon source.* Journal of Bioscience and Bioengineering, 2020. **129**(4): p. 428-434.
69. Pierson, J.T., et al., *Phytochemical extraction, characterisation and comparative distribution across four mango (Mangifera indica L.) fruit varieties.* Food chemistry, 2014. **149**: p. 253-263.
70. Kim, J.-A., et al., *Antioxidant and NF-κB inhibitory constituents isolated from Morchella esculenta.* Natural product research, 2011. **25**(15): p. 1412-1417.
71. Patil, A.D., et al., *Plakortides, novel cyclic peroxides from the sponge Plakortis halichondrioides: activators of cardiac SR-Ca2+-pumping ATPase.* Journal of natural products, 1996. **59**(3): p. 219-223.
72. Otify, A., et al., *Mechanistic evidence of Passiflora edulis (Passifloraceae) anxiolytic activity in relation to its metabolite fingerprint as revealed via LC-MS and chemometrics.* Food & function, 2015. **6**(12): p. 3807-3817.
73. Otify, A.M., et al., *Metabolites profiling of date palm (Phoenix dactylifera L.) commercial by-products (pits and pollen) in relation to its antioxidant effect: a multiplex approach of MS and NMR metabolomics.* Metabolomics, 2019. **15**(9): p. 119.
74. Gillan, F.T., R.W. Hogg, and E.A. Drew, *The sterol and fatty acid compositions of seven tropical seagrasses from North Queensland, Australia.* Phytochemistry, 1984. **23**(12): p. 2817-2821.

75. Vaskovsky, V. and S. Khotimchenko, *Chemotaxonomic approach protects against mistakes in polyunsaturated fatty acid analyses in plants.* Journal of the American Oil Chemists' Society, 1992. **69**(6): p. 598-598.
76. Goncharova, S., N. Sanina, and E. Kostetsky, *Role of lipids in molecular thermoadaptation mechanisms of seagrass Zostera marina*. 2000, Portland Press Ltd.
